# Bis(1-methyl­piperazine-1,4-diium) di-μ-bromido-bis­[tetra­bromido­bismuthate(III)] dihydrate

**DOI:** 10.1107/S1600536814009805

**Published:** 2014-05-10

**Authors:** Manel Essid, Thierry Roisnel, Houda Marouani

**Affiliations:** a Laboratoire de Chimie des Matériaux, Faculté des Sciences de Bizerte, 7021 Zarzouna Bizerte, Tunisia; b Centre de Diffractométrie X, UMR 6226 CNRS, Unité Sciences Chimiques de Rennes, Université de Rennes I, 263 Avenue du Général Leclerc, 35042 Rennes, France

## Abstract

In the title hydrated salt, (C_5_H_14_N_2_)_2_[Bi_2_Br_10_]·2H_2_O, the com­plete [Bi_2_Br_10_]^4−^ biocta­hedron is generated by crystallographic inversion symmetry. The diprotonated piperazine ring adopts a chair conformation, with the methyl group occupying an equatorial position. In the crystal, the tetra­anions and water mol­ecules are linked by O—H⋯Br and O—H⋯(Br,Br) hydrogen bonds to generate [100] chains. The chains are crosslinked by N—H⋯Br, N—H⋯O and C—H⋯Br hydrogen bonds originating from the piperazinediium dications, thereby forming a three-dimensional network.

## Related literature   

For another deca­bromido­dibismuthate(III) compound, see: Li *et al.* (2006 ▶). For related methyl­piperazin-1,4-diium salts, see: Dutkiewicz *et al.* (2011 ▶); Essid *et al.* (2014 ▶). For related piperazine derivatives, see: Marouani *et al.* (2010 ▶); Essid *et al.* (2010 ▶).
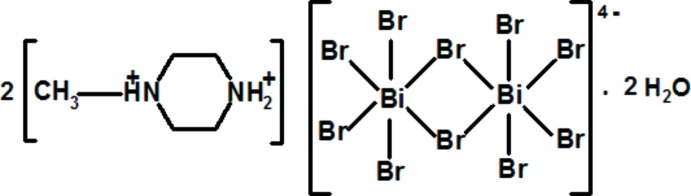


## Experimental   

### 

#### Crystal data  

(C_5_H_14_N_2_)_2_[Bi_2_Br_10_]·2H_2_O
*M* *_r_* = 1457.46
Monoclinic, 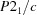

*a* = 7.9263 (3) Å
*b* = 19.0424 (7) Å
*c* = 12.5861 (4) Å
β = 125.770 (2)°
*V* = 1541.35 (9) Å^3^
*Z* = 2
Mo *K*α radiation
μ = 24.38 mm^−1^
*T* = 150 K
0.15 × 0.12 × 0.07 mm

#### Data collection  

Bruker APEXII diffractometer
Absorption correction: multi-scan (*SADABS*; Bruker, 2006 ▶) *T*_min_ = 0.071, *T*_max_ = 0.182
23912 measured reflections
3525 independent reflections
3262 reflections with *I* > 2σ(*I*)
*R*_int_ = 0.051

#### Refinement  

*R*[*F* ^2^ > 2σ(*F* ^2^)] = 0.023
*wR*(*F* ^2^) = 0.056
*S* = 1.09
3525 reflections
136 parameters
3 restraints
H atoms treated by a mixture of independent and constrained refinement
Δρ_max_ = 1.23 e Å^−3^
Δρ_min_ = −1.52 e Å^−3^

### 

Data collection: *APEX2* (Bruker, 2006 ▶); cell refinement: *APEX2*; data reduction: *APEX2*; program(s) used to solve structure: *SIR97* (Altomare *et al.*, 1999 ▶); program(s) used to refine structure: *SHELXL97* (Sheldrick, 2008 ▶); molecular graphics: *ORTEP-3 for Windows* (Farrugia, 2012 ▶) and *DIAMOND* (Brandenburg & Putz, 2005 ▶); software used to prepare material for publication: *WinGX* (Farrugia, 2012 ▶) and *CRYSCAL* (T. Roisnel, local program).

## Supplementary Material

Crystal structure: contains datablock(s) I, global. DOI: 10.1107/S1600536814009805/hb7225sup1.cif

Structure factors: contains datablock(s) I. DOI: 10.1107/S1600536814009805/hb7225Isup2.hkl

CCDC reference: 1000442

Additional supporting information:  crystallographic information; 3D view; checkCIF report

## Figures and Tables

**Table 1:** Selected bond lengths (Å)

Bi—Br3	2.7441 (5)
Bi—Br4	2.7714 (5)
Bi—Br5	2.7730 (5)
Bi—Br2	2.8784 (5)
Bi—Br1	2.9746 (5)
Bi—Br1^i^	3.0056 (5)

Symmetry code: (i) 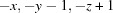.

**Table 2:** Hydrogen-bond geometry (Å, °)

*D*—H⋯*A*	*D*—H	H⋯*A*	*D*⋯*A*	*D*—H⋯*A*
N1—H1⋯Br5	0.91	2.66	3.396 (4)	138
N1—H1⋯Br2	0.91	2.85	3.486 (4)	128
N2—H2*C*⋯O	0.90	1.91	2.793 (6)	167
N2—H2*D*⋯Br2^ii^	0.90	2.60	3.371 (4)	143
O—H2⋯Br5^iii^	0.94 (1)	2.91 (7)	3.522 (4)	123 (6)
O—H2⋯Br1^iii^	0.94 (1)	2.84 (7)	3.531 (4)	131 (7)
O—H3⋯Br3^iv^	0.95 (1)	2.59 (2)	3.501 (4)	162 (6)
C2—H2*B*⋯Br2^v^	0.97	2.80	3.649 (5)	147

Symmetry codes: (ii) 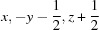; (iii) 
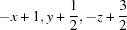
; (iv) 
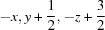
; (v) 
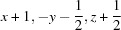
.

**Fig. 1.**
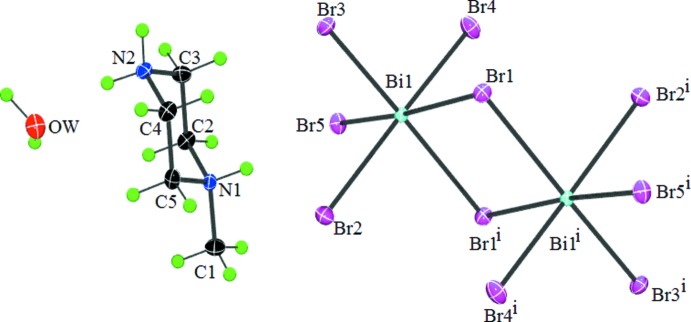
An ORTEP view of (I) with displacement ellipsoids drawn at the 50% probability level. Symmetry code: i: -x, -y, -z.

**Fig. 2.**
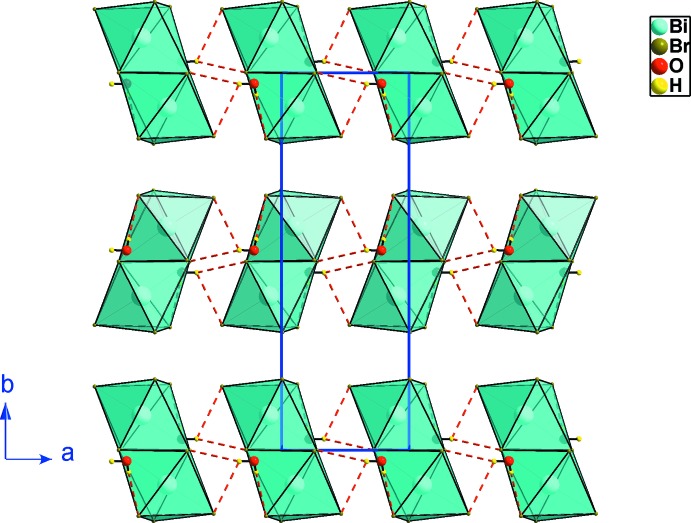
Projection of the corrugated inorganic chains along the c axis.

**Fig. 3.**
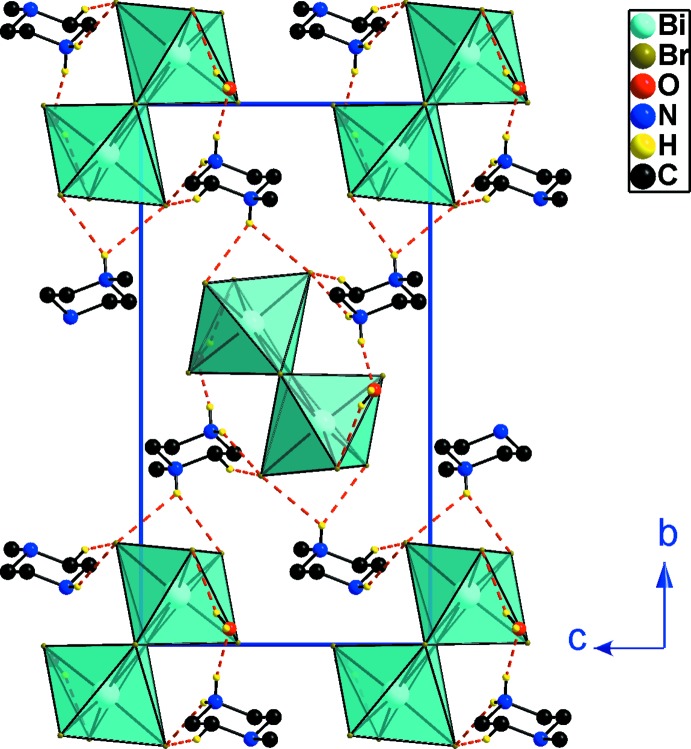
Projection of (I) along the a axis. The H-atoms not involved in H-bonding are omitted.

## supplementary crystallographic information

### 1. Comment

As a part of our study of crystal packing containing piperazine derivatives (Marouani *et al.*, 2010; Essid *et al.*, 2010; Essid *et al.*, 2014), we report here the preparation and the structural investigation of a new compound, (C_5_H_14_N_2_)_2_Bi_2_Br_10_·2H_2_O, (I)

The crystal structure of the title compound (I) is built up of two 1-methylpiperazinium dications, two water molecules and decabromodibismuthate tetraanions; the latter have two octahedra sharing a common edge and occupy special positions with a centre of symmetry at the centre of the Bi_2_Br_2_ ring. Its geometrical configuration is depicted in figure 1. The half of this formula constitutes the asymmetric unit in the atomic arrangement. In the title compound (I) the [Bi_2_Br_10_]^4-^ bioctahedra anions are connected through O–H···Br hydrogen bonds (*via* water molecules) and form infinite unidimensional chains of composition [Bi_2_Br_10_(H_2_O)_2_]_n_^4n-^ parallel to the *a* axis (Fig.2). These chains are themselves interconnected by means of N–H···Br, N–H···O and C–H···Br bonds originating from the [C_5_H_14_N_2_]^2+^ entities, forming a three-dimensional network (Fig. 3). The coordination octahedral of the bismuth atoms are formed by six bromine atoms, as shown in Fig. 1. The Bi–Br distances listed in Table 1 vary from 2.7441 (5) to 3.0056 (5) Å with mean value of 2.8579 Å. The Br–Bi–Br angles range from 85.170 (15)° to 99.774 (14)° and from 170.436 (15)° to 173.695 (15)°, indicating that the BiBr_6_ octahedron is distorted. These values are in agreement with those commonly observed in other organic decabromodibismuthate(III) compound (Li *et al.*, 2006). Geometrical parameters of the methylpiperazin-1,4-dium dications are found to be in agreement with those reported in related methylpiperazin-1,4-diium salts (Dutkiewicz *et al.*, 2011; Essid *et al.*, 2014). The cyclic amine adopts a chair conformation with the methyl group occupying an equatorial position, with puckering parameters: Q = 0.573 (5) Å, θ = 0.8 (5)° and φ = 116 (4)° and atoms N1 and N2 deviating by -0.314 and 0.322 Å from the least-squares plane defined by the remaining atoms in the ring. The cations are linked onto the anionic chains, by forming H-bonds with the bromine and oxygen atoms with donor-acceptor distances in the range 2.793 (6)–3.649 (5) Å (Table 2).

### 2. Experimental

Bismuth(III) nitrate and 1-(methyl) piperazine were dissolved in a concentrated HBr solution in the presence of ethanol (40 ml) and water (20 ml) in a stoichiometric ratio. Colourless prisms of the title compound were obtained by slow evaporation of this solution at room temperature.

### 3. Refinement

The hydrogen atoms bonded to oxygen atoms were located from a difference map and were allowed to refine using restraints [O—H = 0.95 (1) A °, H···H = 1.44 (2) A ° and *U*_iso_(H) = 1.5*U*_eq_(O)]. The rest of the H atoms were treated as riding, with C—H = 0.96 Å (methyl), or C—H = 0.97 Å (methylene), or N—H = 0.91 Å (NH), or N—H = 0.90 Å (NH_2_) with *U*_iso_(H) = 1.2*U*_eq_(C or N).

### Crystal data

**Table:**

(C_5_H_14_N_2_)_2_[Bi_2_Br_10_]·2H_2_O	*F*(000) = 1304
*M**_r_* = 1457.46	*D*_x_ = 3.140 Mg m^−^^3^
Monoclinic, *P*2_1_/*c*	Mo *K*α radiation, λ = 0.71073 Å
Hall symbol: -P 2ybc	Cell parameters from 9944 reflections
*a* = 7.9263 (3) Å	θ = 2.8–27.5°
*b* = 19.0424 (7) Å	µ = 24.38 mm^−^^1^
*c* = 12.5861 (4) Å	*T* = 150 K
β = 125.770 (2)°	Prism, colourless
*V* = 1541.35 (9) Å^3^	0.15 × 0.12 × 0.07 mm
*Z* = 2	

### Data collection

**Table:**

Bruker APEXII diffractometer	3262 reflections with *I* > 2σ(*I*)
Graphite monochromator	*R*_int_ = 0.051
CCD rotation images, thin slices scans	θ_max_ = 27.5°, θ_min_ = 2.9°
Absorption correction: multi-scan (*SADABS*; Bruker, 2006)	*h* = −10→10
*T*_min_ = 0.071, *T*_max_ = 0.182	*k* = −22→24
23912 measured reflections	*l* = −13→12
3525 independent reflections	

### Refinement

**Table:**

Refinement on *F*^2^	Primary atom site location: structure-invariant direct methods
Least-squares matrix: full	Secondary atom site location: difference Fourier map
*R*[*F*^2^ > 2σ(*F*^2^)] = 0.023	Hydrogen site location: inferred from neighbouring sites
*wR*(*F*^2^) = 0.056	H atoms treated by a mixture of independent and constrained refinement
*S* = 1.09	*w* = 1/[σ^2^(*F*_o_^2^) + (0.0151*P*)^2^ + 6.909*P*] where *P* = (*F*_o_^2^ + 2*F*_c_^2^)/3
3525 reflections	(Δ/σ)_max_ = 0.041
136 parameters	Δρ_max_ = 1.23 e Å^−^^3^
3 restraints	Δρ_min_ = −1.52 e Å^−^^3^

### Special details

**Table:**

Geometry. All e.s.d.'s (except the e.s.d. in the dihedral angle between two l.s. planes) are estimated using the full covariance matrix. The cell e.s.d.'s are taken into account individually in the estimation of e.s.d.'s in distances, angles and torsion angles; correlations between e.s.d.'s in cell parameters are only used when they are defined by crystal symmetry. An approximate (isotropic) treatment of cell e.s.d.'s is used for estimating e.s.d.'s involving l.s. planes.
Refinement. Refinement of *F*^2^ against ALL reflections. The weighted *R*-factor *wR* and goodness of fit *S* are based on *F*^2^, conventional *R*-factors *R* are based on *F*, with *F* set to zero for negative *F*^2^. The threshold expression of *F*^2^ > σ(*F*^2^) is used only for calculating *R*-factors(gt) *etc*. and is not relevant to the choice of reflections for refinement. *R*-factors based on *F*^2^ are statistically about twice as large as those based on *F*, and *R*- factors based on ALL data will be even larger.

### Fractional atomic coordinates and isotropic or equivalent isotropic displacement parameters (Å^2^)

**Table:**

	*x*	*y*	*z*	*U*_iso_*/*U*_eq_	
Bi	0.10118 (2)	−0.407806 (8)	0.622821 (15)	0.01138 (6)	
Br1	0.27752 (7)	−0.49972 (2)	0.51922 (4)	0.01697 (10)	
Br2	−0.00499 (8)	−0.31232 (3)	0.41496 (5)	0.02036 (11)	
Br3	−0.12297 (8)	−0.32553 (3)	0.67787 (5)	0.02123 (11)	
Br4	0.25521 (8)	−0.49702 (3)	0.83467 (5)	0.02381 (12)	
Br5	0.46641 (7)	−0.33162 (2)	0.77789 (5)	0.02014 (11)	
O	0.2175 (6)	0.02902 (19)	0.6937 (4)	0.0248 (8)	
N1	0.3077 (6)	−0.1749 (2)	0.6194 (4)	0.0145 (8)	
H1	0.2760	−0.2201	0.6249	0.017*	
C1	0.4166 (8)	−0.1760 (3)	0.5543 (5)	0.0235 (11)	
H1A	0.3234	−0.1933	0.4665	0.035*	
H1B	0.5361	−0.2061	0.6025	0.035*	
H1C	0.4600	−0.1292	0.5522	0.035*	
C2	0.4472 (7)	−0.1466 (3)	0.7560 (4)	0.0167 (9)	
H2A	0.4885	−0.0990	0.7534	0.020*	
H2B	0.5718	−0.1751	0.8064	0.020*	
C3	0.3371 (8)	−0.1467 (3)	0.8219 (5)	0.0195 (10)	
H3A	0.3068	−0.1946	0.8317	0.023*	
H3B	0.4269	−0.1261	0.9085	0.023*	
N2	0.1400 (6)	−0.1059 (2)	0.7427 (4)	0.0168 (8)	
H2C	0.1693	−0.0607	0.7387	0.020*	
H2D	0.0746	−0.1073	0.7818	0.020*	
C4	0.0000 (7)	−0.1347 (3)	0.6073 (5)	0.0184 (10)	
H4A	−0.1254	−0.1065	0.5570	0.022*	
H4B	−0.0397	−0.1824	0.6109	0.022*	
C5	0.1080 (7)	−0.1344 (2)	0.5413 (5)	0.0177 (10)	
H5A	0.0174	−0.1550	0.4548	0.021*	
H5B	0.1368	−0.0863	0.5310	0.021*	
H2	0.360 (4)	0.035 (4)	0.730 (7)	0.08 (3)*	
H3	0.203 (10)	0.062 (3)	0.745 (6)	0.05 (2)*	

### Atomic displacement parameters (Å^2^)

**Table:**

	*U*^11^	*U*^22^	*U*^33^	*U*^12^	*U*^13^	*U*^23^
Bi	0.00972 (9)	0.01144 (9)	0.01281 (9)	−0.00006 (6)	0.00649 (7)	−0.00024 (6)
Br1	0.0124 (2)	0.0197 (2)	0.0184 (2)	0.00040 (17)	0.0087 (2)	−0.00129 (17)
Br2	0.0198 (3)	0.0218 (2)	0.0206 (2)	0.00041 (19)	0.0125 (2)	0.00260 (18)
Br3	0.0254 (3)	0.0199 (2)	0.0242 (2)	0.00474 (19)	0.0178 (2)	−0.00034 (18)
Br4	0.0202 (3)	0.0235 (3)	0.0190 (2)	−0.00190 (19)	0.0065 (2)	0.00602 (19)
Br5	0.0143 (2)	0.0189 (2)	0.0224 (2)	−0.00246 (18)	0.0080 (2)	−0.00086 (18)
O	0.0156 (18)	0.0231 (19)	0.028 (2)	−0.0021 (15)	0.0085 (16)	0.0002 (15)
N1	0.016 (2)	0.0114 (18)	0.018 (2)	−0.0006 (15)	0.0112 (17)	−0.0011 (15)
C1	0.026 (3)	0.026 (3)	0.029 (3)	−0.001 (2)	0.022 (2)	0.000 (2)
C2	0.010 (2)	0.021 (2)	0.015 (2)	−0.0014 (18)	0.0052 (19)	−0.0039 (18)
C3	0.019 (2)	0.024 (3)	0.016 (2)	0.001 (2)	0.010 (2)	0.0007 (19)
N2	0.018 (2)	0.017 (2)	0.018 (2)	0.0023 (16)	0.0122 (18)	−0.0001 (16)
C4	0.013 (2)	0.021 (2)	0.019 (2)	0.0005 (19)	0.008 (2)	−0.0023 (19)
C5	0.016 (2)	0.016 (2)	0.013 (2)	0.0031 (18)	0.0036 (19)	0.0008 (17)

### Geometric parameters (Å, º)

**Table:**

Bi—Br3	2.7441 (5)	C1—H1C	0.9600
Bi—Br4	2.7714 (5)	C2—C3	1.515 (7)
Bi—Br5	2.7730 (5)	C2—H2A	0.9700
Bi—Br2	2.8784 (5)	C2—H2B	0.9700
Bi—Br1	2.9746 (5)	C3—N2	1.489 (6)
Bi—Br1^i^	3.0056 (5)	C3—H3A	0.9700
Br1—Bi^i^	3.0056 (5)	C3—H3B	0.9700
O—H2	0.943 (10)	N2—C4	1.492 (6)
O—H3	0.945 (10)	N2—H2C	0.9000
N1—C1	1.497 (6)	N2—H2D	0.9000
N1—C2	1.500 (6)	C4—C5	1.501 (7)
N1—C5	1.500 (6)	C4—H4A	0.9700
N1—H1	0.9100	C4—H4B	0.9700
C1—H1A	0.9600	C5—H5A	0.9700
C1—H1B	0.9600	C5—H5B	0.9700
			
Br3—Bi—Br4	95.349 (16)	N1—C2—H2A	109.5
Br3—Bi—Br5	95.001 (16)	C3—C2—H2A	109.5
Br4—Bi—Br5	87.249 (15)	N1—C2—H2B	109.5
Br3—Bi—Br2	88.872 (15)	C3—C2—H2B	109.5
Br4—Bi—Br2	172.766 (16)	H2A—C2—H2B	108.1
Br5—Bi—Br2	86.531 (15)	N2—C3—C2	110.3 (4)
Br3—Bi—Br1	170.436 (15)	N2—C3—H3A	109.6
Br4—Bi—Br1	90.312 (15)	C2—C3—H3A	109.6
Br5—Bi—Br1	92.961 (15)	N2—C3—H3B	109.6
Br2—Bi—Br1	86.326 (14)	C2—C3—H3B	109.6
Br3—Bi—Br1^i^	85.170 (15)	H3A—C3—H3B	108.1
Br4—Bi—Br1^i^	86.460 (14)	C3—N2—C4	111.2 (4)
Br5—Bi—Br1^i^	173.695 (15)	C3—N2—H2C	109.4
Br2—Bi—Br1^i^	99.774 (14)	C4—N2—H2C	109.4
Br1—Bi—Br1^i^	87.498 (13)	C3—N2—H2D	109.4
Bi—Br1—Bi^i^	92.502 (13)	C4—N2—H2D	109.4
H2—O—H3	100 (2)	H2C—N2—H2D	108.0
C1—N1—C2	111.0 (4)	N2—C4—C5	110.3 (4)
C1—N1—C5	111.9 (4)	N2—C4—H4A	109.6
C2—N1—C5	110.8 (3)	C5—C4—H4A	109.6
C1—N1—H1	107.7	N2—C4—H4B	109.6
C2—N1—H1	107.7	C5—C4—H4B	109.6
C5—N1—H1	107.7	H4A—C4—H4B	108.1
N1—C1—H1A	109.5	N1—C5—C4	111.1 (4)
N1—C1—H1B	109.5	N1—C5—H5A	109.4
H1A—C1—H1B	109.5	C4—C5—H5A	109.4
N1—C1—H1C	109.5	N1—C5—H5B	109.4
H1A—C1—H1C	109.5	C4—C5—H5B	109.4
H1B—C1—H1C	109.5	H5A—C5—H5B	108.0
N1—C2—C3	110.7 (4)		

Symmetry code: (i) −*x*, −*y*−1, −*z*+1.

### Hydrogen-bond geometry (Å, º)

**Table:**

*D*—H···*A*	*D*—H	H···*A*	*D*···*A*	*D*—H···*A*
N1—H1···Br5	0.91	2.66	3.396 (4)	138
N1—H1···Br2	0.91	2.85	3.486 (4)	128
N2—H2*C*···O	0.90	1.91	2.793 (6)	167
N2—H2*D*···Br2^ii^	0.90	2.60	3.371 (4)	143
O—H2···Br5^iii^	0.94 (1)	2.91 (7)	3.522 (4)	123 (6)
O—H2···Br1^iii^	0.94 (1)	2.84 (7)	3.531 (4)	131 (7)
O—H3···Br3^iv^	0.95 (1)	2.59 (2)	3.501 (4)	162 (6)
C2—H2*B*···Br2^v^	0.97	2.80	3.649 (5)	147

Symmetry codes: (ii) *x*, −*y*−1/2, *z*+1/2; (iii) −*x*+1, *y*+1/2, −*z*+3/2; (iv) −*x*, *y*+1/2, −*z*+3/2; (v) *x*+1, −*y*−1/2, *z*+1/2.
